# Dancing or Fitness Sport? The Effects of Two Training Programs on Hippocampal Plasticity and Balance Abilities in Healthy Seniors

**DOI:** 10.3389/fnhum.2017.00305

**Published:** 2017-06-15

**Authors:** Kathrin Rehfeld, Patrick Müller, Norman Aye, Marlen Schmicker, Milos Dordevic, Jörn Kaufmann, Anita Hökelmann, Notger G. Müller

**Affiliations:** ^1^German Center for Neurodegenerative DiseasesMagdeburg, Germany; ^2^Institute for Sport Science, Otto von Guericke University MagdeburgMagdeburg, Germany; ^3^Medical Faculty, Otto von Guericke University MagdeburgMagdeburg, Germany; ^4^Department of Neurology, Otto von Guericke University MagdeburgMagdeburg, Germany; ^5^Center for Behavioral Brain SciencesMagdeburg, Germany

**Keywords:** dancing, fitness training, balance, hippocampus, aging

## Abstract

Age-related degenerations in brain structure are associated with balance disturbances and cognitive impairment. However, neuroplasticity is known to be preserved throughout lifespan and physical training studies with seniors could reveal volume increases in the hippocampus (HC), a region crucial for memory consolidation, learning and navigation in space, which were related to improvements in aerobic fitness. Moreover, a positive correlation between left HC volume and balance performance was observed. Dancing seems a promising intervention for both improving balance and brain structure in the elderly. It combines aerobic fitness, sensorimotor skills and cognitive demands while at the same time the risk of injuries is low. Hence, the present investigation compared the effects of an 18-month dancing intervention and traditional health fitness training on volumes of hippocampal subfields and balance abilities. Before and after intervention, balance was evaluated using the Sensory Organization Test and HC volumes were derived from magnetic resonance images (3T, MP-RAGE). Fourteen members of the dance (67.21 ± 3.78 years, seven females), and 12 members of the fitness group (68.67 ± 2.57 years, five females) completed the whole study. Both groups revealed hippocampal volume increases mainly in the left HC (CA1, CA2, subiculum). The dancers showed additional increases in the left dentate gyrus and the right subiculum. Moreover, only the dancers achieved a significant increase in the balance composite score. Hence, dancing constitutes a promising candidate in counteracting the age-related decline in physical and mental abilities.

## Introduction

The human hippocampus (HC) is affected not only by pathological aging such as in Alzheimer’s disease but also by the normal aging process resulting in deficits in memory, learning, and spatial navigation at old age (Driscoll et al., 2003; Barnes et al., 2009). Magnetic resonance-studies indicate an atrophy rate of the hippocampus and the nearby parahippocampal gyrus of 2–3% per decade (Raz et al., 2004, 2005), which is further accelerated in the very old age where there is an annual loss of 1% over the age of 70 (Jack et al., 1998). On the other hand more recent research has shown that the HC counts among the few brain regions with the ability to generate new neurons throughout the lifespan (Kempermann et al., 2010; Spalding et al., 2013). In animal models physical activity has been identified as a key mechanism that can drive this adult neuroplasticity (van Praag et al., 1999; Kronenberg, 2003). In humans, research has focused on the effects of aerobic fitness and training on volumes and perfusion of the HC. Results reveal that higher cardiorespiratory fitness levels (VO_2_ max) are associated with larger hippocampal volumes in late adulthood, and that larger hippocampal volumes may, in turn, contribute to better memory function (Erickson et al., 2011; Szabo et al., 2011; Bugg et al., 2012; Maass et al., 2015). Furthermore, some investigations also assessed possible physiological mediators of the observed neuroplasticity, such as brain-derived neurotrophic factor (BDNF), insulin-like growth factor 1 (IGF-1), and vascular endothelial growth factor (VEGF) (Flöel et al., 2010; Erickson et al., 2011; Ruscheweyh et al., 2011; Maass et al., 2016). Whereas Erickson et al. (2011) reported a positive correlation between levels of serum BDNF, hippocampal volume and cardiorespiratory fitness during 1 year of aerobic training, neither Ruscheweyh et al. (2011) nor Maass et al. (2016) found fitness-related BDNF changes after 6 or 3 months of training, respectively. Moreover, other studies failed to find correlations between volumes of the medial temporal lobe area or the hippocampus and cardiovascular fitness in healthy elderly (Honea et al., 2009; Smith et al., 2011). Therefore, the role of cardio-respiratory fitness in modulating hippocampal gray matter volume is still under debate.

The hippocampus is also involved in spatial navigation (O’Keefe, 1990) and in motor sequence consolidation (Albouy et al., 2008) suggesting that motor skill learning and motor fitness can have impact on hippocampal volume without any cardio-respiratory change. In this respect, Niemann et al. (2014) tested whether 12 months of cardiovascular or coordination training induces larger increases in hippocampal volume in healthy older participants. After training, the cardio-vascular group revealed a significant volume increase in the left HC of 4.22% and a non-significant increase of 2.98% for the right HC. Effects of the coordinative training were more pronounced in the right HC with an increase of 3.91%, whereas the changes in the left HC (1.78%) were non-significant. Further correlation analyses between motor fitness and hippocampal volume failed to reach significant results. Still there is compelling evidence that the human brain undergoes morphological alterations in response to motor-skill learning (Draganski et al., 2004; Boyke et al., 2008; Taubert et al., 2010; Sehm et al., 2014). Along these lines, a recent study demonstrated structural brain changes already after two sessions of dynamic balance training that correlated with the individual motor skill learning success of the participants (Taubert et al., 2010). Sehm et al. (2014) could demonstrate that 6 weeks of balance training induced increases in the gray matter of the left HC in healthy seniors. These findings highlight the behavioral relevance of structural brain plasticity in the HC for the learning process. Hüfner et al. (2011) stated that long-term balance training with its extensive vestibular, visual and sensorimotor stimulation is associated with altered hippocampal formation volumes in professional ballet dancers and slackliners. Hence, the HC seems not only crucial for long-term memory consolidation, learning and spatial navigation, but also for balancing. Intact balance is essential for social mobility and quality of life in aging (Dordevic et al., 2017). Hence, physical intervention programs should take this function into account, too.

In this respect dancing seems to be a promising intervention since it requires the integration of sensory information from multiple channels (auditory, vestibular, visual, somatosensory) and the fine-grained motor control of the whole body. Behavioral studies have already provided evidence of better performance in balance and memory tasks in elderly dancers (Kattenstroth et al., 2010, 2013; Rehfeld et al., 2014), but the underlying neural mechanisms have not been addressed comprehensively so far. Knowing that aerobic, sensorimotor and cognitive training contribute to hippocampal volume, which also seems to be associated with balancing capabilities, we initialized a prospective, randomized longitudinal trial over a period of 18 months in healthy seniors. Two interventions were compared: a specially designed dance program, during which subjects constantly had to learn new choreographies, and a traditional fitness program with mainly repetitive exercises, such as cycling on an ergometer or Nordic walking. Whole-brain analyzes of the acquired data using voxel based morphometry had shown dance-associated volume increases mainly in the precentral and the parahippocampal gyrus (Müller et al., 2016). Knowing that dancing/slacklining (Hüfner et al., 2011) and endurance sport (e.g., Erickson et al., 2011) have different impact on anterior and posterior parts of the hippocampus in the present analysis we ran a region of interest analysis of this specific brain region. To do so we first computed a restricted VBM analysis with a hippocampal mask. In the next step we divided the hippocampus in five subfields in order to allow a detailed analysis of the interventions’ effects on different parts of the HC. The hippocampus is not a homogeneous structure but consists of histologically specialized subfields, such as the subiculum, cornu ammonis (CA) 1–4 and dentate gyrus (DG). The subiculum has been implicated in working memory and spatial relations (Riegert et al., 2004; O’Mara, 2005). CA3 and DG have been suggested to be involved in memory and early retrieval, whereas CA1 in late retrieval, consolidation and recognition. Especially the DG is one of the few regions of the adult brain where neurogenesis takes places, which is important in the formation of new memories and spatial memory (Saab et al., 2009). Nevertheless all these subfields are tightly interconnected (Duvernoy, 2005). Since dancing seems to promote spatial orientation, working memory and might promote neurogenesis, we expected volume changes in more subfields of the HC after this intervention. Moreover, given the importance of intact balance for successful aging on the one hand and its dependence on the hippocampus on the other hand, we also assessed effects of the interventions on balancing capabilities and their relation to hippocampal subfield volumes.

## Materials and Methods

### Study Design and Subjects

**FIGURE 1:**
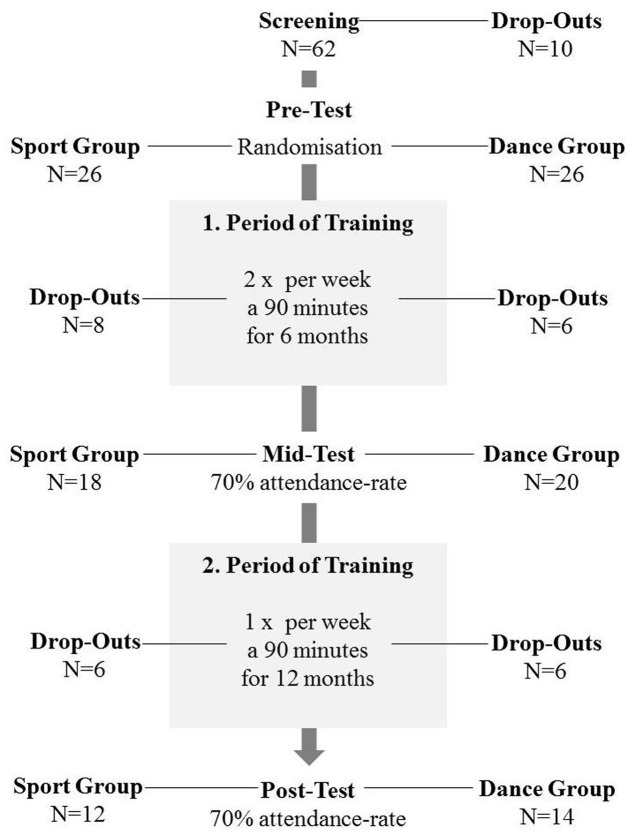
Study design presenting: voluntary recruitment, drop-outs, time-points of measurement, duration of training periods.

This investigation, comprising hippocampal volume alterations and changes in balance abilities, is part of a large prospective longitudinal study which compares the effects of dancing versus aerobic training on brain structure and function, mediating neuroplasticity factors, such as BDNF, as well as cognitive and motor performances in healthy elderly seniors. The cognitive development and BDNF changes are highlighted in our recent report (see Müller et al., 2017). The intervention was provided for 18 months and contained three time-points of measurement: baseline pre-test, first post-test after 6 months of training and second post-test after 18 months of training (see **Figure 1**). Again, the temporal dynamics of gray matter brain plasticity are already stated by Müller et al. (2017), showing a significant increase of gray matter volume in parahippocampal gyrus only for the dancers. Based on that finding, we assume only changes from baseline to the second post-test (18 months).

The approval for the study was obtained from the ethics committee of the Otto von Guericke University, Magdeburg. All subjects signed a written informed consent and received a reimbursement for their participation.

The timeline of the study can be depicted from **Figure 1**. Primarily, we invited 62 healthy elderly volunteers aged 63–80 years for cognitive and physical screening as well as for verification of magnetic resonance imaging suitability. Exclusion criteria were defined as follows: any history of severe neurological conditions, metal implants, claustrophobia, tinnitus, intensive physical engagement (more than 1 h/week), cognitive impairments as evidenced in the MMSE (Folstein et al., 1975) and depressive symptoms (BDI-II > 13) (Beck et al., 2006). Fifty two seniors met the inclusion criteria and were then randomly assigned to the experimental dance group and the control sport group. After 18 months of training we were left with 26 complete data sets, including 14 dancers and 12 sportsmen. Both groups (mean age = 67.9 ± 3.3 years) did not differ concerning age, sex, education, and BMI. For detailed information about demographic data see **Table 1**.

**Table 1:** Demographic information at baseline of analyzed participants (*N* = 26).

	Dance group [*N* = 14]		Sport group [*N* = 12]			
	*M*	*SD*	*M*	*SD*	*t*-value	*p*-value
Age [years]	67.21	3.78	68.67	2.57	-1.12	0.272
Sex [%]	50% male		58% male		0.154	0.695
MMSE [points]	28.07	0.92	29.17	0.58	-3.011	0.003
BDI-II [points]	4.5/5.54	3.37/2.97	3.75/3.39	3.79/3.77	0.53	0.598
Education [years]	15.40	2.05	16.10	1.45	-1.395	0.175
BMI [kg/m^2^]	27.51	3.87	27.24	2.94	-0.275	0.786

*BMI, Body-Mass-Index; BDI-II, Becks-Depressions-Inventar II; MMSE, Mini Mental State Examination; M, Mean; SD, Standard deviation; p ≤ 0.05 = statistical significance.*

### Interventions

The precise description of the interventions is published elsewhere (Müller et al., 2016). In brief, the first period of training was provided for 6 months, twice a week for both groups. Each dancing or fitness class lasted 90 min. Because of organizational reasons we had to change the training frequency from twice a week to once a week after 6 months of training. The second training period was run for 12 months and the training sessions were reduced to once a week for 90 min in both groups. The content of the dance classes induced a permanent learning situation with constantly changing choreographies, which participants had to memorize accurately. The training focused on elementary longitudinal turns, head-spins, shifts of center of gravity (COG), single-leg stances, skips and hops, different steps like chassée, mambo, cha cha, grapevine, jazz square to challenge the balance system. Additional arm-patterns enforced imbalances (moving arms away from center of pressure).

The program for the sport group was adjusted according to the recommended guidelines for health sport (Brehm et al., 2006) and included endurance training, strength-endurance training, and flexibility training (stretching and mobility). Each part of the mentioned topics (endurance; strength-endurance; and flexibility) was exercised for 20 min, whereby a 10 min warm-up, a 10 min cool-down and short breaks between the different exercises adding to another 10 min completed each 90 min lasting session. So both groups exercised for 90 min in each training session. In the first 6 months, endurance training was performed on bicycle ergometers with the intensity adjusted to the individual training heart rate (HR) using the Karvonen Formula:

Target training HR = Resting HR + (0.6[maximum HR - resting HR]).

The factor 0.6 is a representative for an extensive aerobic training (Davis and Convertino, 1975). In the second training period (12 months) the participants completed a Nordic Walking program. The strength-endurance training aimed to strengthen major muscles of the muscular skeleton. In this program we avoided combined arm and leg movements in order to keep coordinative demands low.

### Structural MRI Acquisition, Preprocessing, and Analysis

Magnetic resonance (MR) images were acquired on a 3 Tesla Siemens MAGNETOM Verio (Syngo MR B17) using a 32-channel head coil. T-1 weighted MPRAGE sequence (224 sagital slices, voxel size: 0.8 mm × 0.8 mm × 0.8 mm, TR: 2500 ms, TE: 3.47 ms, TI: 1100 ms, flip angle: 7°) were analyzed using region of interest (ROI) defined voxel-based morphometry with SPM 12 (Welcome Department of Cognitive Neurology, London, United Kingdom) running under Matlab (The Math Works). The data preprocessing involved gray matter segmentation, DARTEL based template creation, spatial normalization to MNI-Space and an 5 mm smoothing with a Gaussian kernel as previously described.

### Voxel-Based Morphometry with Hippocampal Mask

In order to incorporate our *a priori* hypotheses concerning hippocampal gray matter volume changes we first conducted a ROI-VBM with hippocampal masks. The longitudinal analysis for hippocampal gray matter volume changes was performed using repeated measurement ANOVAs in a full factorial design. We applied a threshold of *p* < 0.05 (FDR corrected).

### Hippocampal Subfield Volume Measurements

**FIGURE 2:**
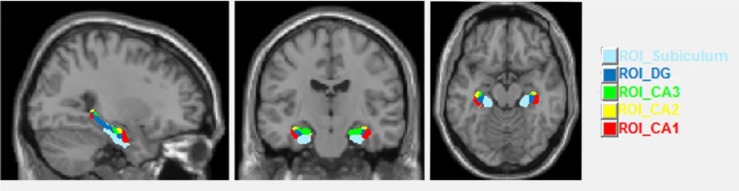
Sagittal, coronal and axial views of the hippocampal subfields.

In a second step we analyzed volume changes in five subfields of the HC. Up to now there is no real gold standard in analyzing HC subfield volumes and each of the current manifold analytic techniques has its strengths and weaknesses (Bandettini, 2009; Kuhnt et al., 2013). Here for the hippocampal subfield segmentation in order to obtain ROI volumes we chose the SPM ANATOMY Toolbox v.2.2.c (Eickhoff et al., 2007) with normalized images. This segmentation included the cornu ammonis (CA1–CA3), the dentate gyrus (DG, including CA4) and the subiculum (**Figure 2**). In SPM Anatomy toolbox, definition of anatomical regions is based on maximum probability cytoarchitectonic maps.

### Postural Control

Postural control was assessed with the Sensory Organization Test (SOT) implemented in the Balance Master System (Neurocom International, Inc., United States). This test provides information about the contribution of the visual, somatosensory, and vestibular system to the maintenance of balance. The system consists of a dual force platform including force transducers measuring the angular displacement of the COG under certain conditions and visual surround. Both, visual surround and platform enable anterior/posterior sway and this sway can be assessed under different conditions. The six conditions are: normal vision and fixed support (condition 1), absent vision and fixed support (condition 2), sway-referenced vision and fixed support (condition 3), normal vision and sway-referenced support (condition 4), absent vision and sway referenced support (condition 5), and sway-referenced vision and sway-referenced support (condition 6). These conditions were performed in three trials for 20 s, resulting in equilibrium scores. Those equilibrium scores range from 0% (balance loss) to 100% (perfect stability). From the equilibrium scores a sensory analysis was performed by calculating average scores of specific pairs of SOT conditions: the participant’s ability to use input from the somatosensory system to maintain balance is reflected by the average of condition 2 divided by the average of condition 1, the contribution of the visual system by the average of condition 4 divided by the average of condition 1 and that of the vestibular system by the average of condition 5 divided by the average of condition 1.

The composite score was calculated by averaging the score for conditions 1 and 2; adding these two scores to the equilibrium scores from each trial of sensory conditions 3, 4, 5, and 6; and dividing that sum by the total number of trials (NeuroCom Natus Medical Incorporated, 2008).

### Statistical Analysis

Statistical analysis of hippocampal volumes and balance data were performed with SPSS (SPSS 22, inc./IBM). Intervention effects were tested using repeated-measurement ANOVAs with group (dance, sport) as between-subject factor and time (pre, post) as within-subject factor. Hereby, age, gender, and total hippocampal volume were included as covariates. Additionally, hypothesis driven *t*-tests (with Bonferroni adjustment) were performed to determine longitudinal changes in the dance and the sports group separately. In case of missing normal distribution we used the Mann–Whitney-*U*-test or Wilcoxon instead of *t*-tests. Pearson-Correlation analysis was performed between percentage change of hippocampal subfield volumes and the balance composite score.

## Results

The presentation of the results is structured as follows. We first tested for hippocampal volume differences after intervention using both masked VBM and subfield volume measurements. In the next step we investigated balance data and finally we looked for correlations between improvements in balance and hippocampal volume.

### Voxel-Based Morphometry with Hippocampal Mask

**FIGURE 3:**
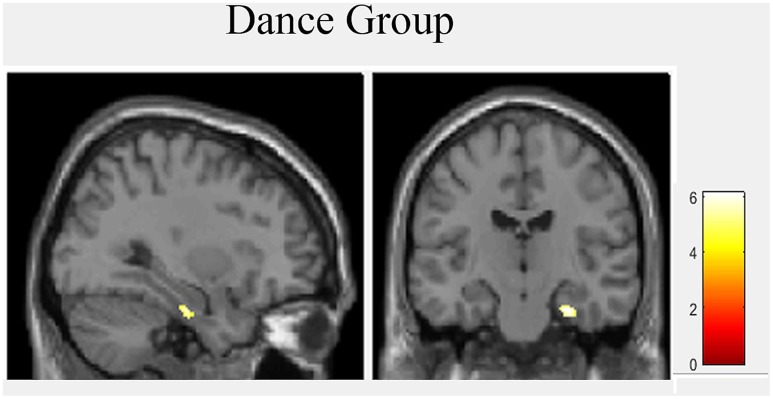
Hippocampal volume increases after 18 month of intervention in dance group.

A two-sample *t*-test revealed no group differences at baseline. To explore hippocampal gray matter volume changes during intervention we used repeated measurement ANOVA for comparison between baseline and post-test. There was a significant interaction effect in the right hippocampus [MNI-coordinates: *x* = 28, *y* = -16, *z* = -23; *p*(FDR) = 0.049, *F* = 17.03]. *Post hoc* paired *t*-tests showed only in the dance group significant volume increases in the right hippocampus [MNI-coordinates: *x* = 29, *y* = -16, *z* = -27; *p*(FDR) = 0.001, *t* = 6.10] (**Figure 3**).

### Hippocampal Subfield Volume Measurements

**FIGURE 4:**
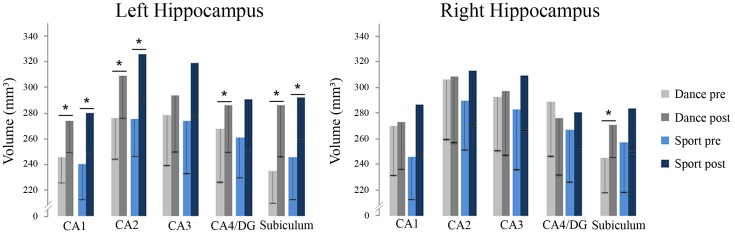
Volumes of hippocampal subfields in dance and sport group at baseline and after 18 months of intervention including standard deviation (DG, dentate gyrus; CA, cornu ammonis; ^∗^*p* < 0.05).

A two-sample *t*-test revealed no group differences of total hippocampal volumes at baseline [*t*(25) = -1.078, *p* = 0.658, *d* = -0,424]. The repeated measurement ANOVA of hippocampal subfield volumes showed a main effect of time regarding left CA1, left CA2, left and right subiculum and left CA4/dentate gyrus (**Table 2**). There were no significant interactions with group. Paired *t*-tests showed significant volume increases for the dancers in left CA1, left CA2, left CA4/dentate gyrus and left and right subiculum and for the sportsmen in the left CA1, left CA2, and left subiculum (**Figure 4**).

**Table 2:** Statistical values of repeated-measures ANOVAs for hippocampal subfields.

Hippocampal subfield	Main effect of time				Main effect of group				Interaction (time × group)			
	*F*	df	*p*	η^2^	*F*	df	*p*	η^2^	*F*	df	*p*	η^2^
Left CA1	16.920	1.25	**0.001^∗∗∗^**	0.413	0.000	1.25	0.985	0.000	0.469	1.25	0.500	0.019
Right CA1	2.952	1.25	0.099	0.110	0.116	1.25	0.736	0.005	2.189	1.25	0.152	0.084
Left CA2	21.126	1.25	**0.001^∗∗∗^**	0.468	0.427	1.25	0.520	0.017	0.941	1.25	0.342	0.038
Right CA2	2.020	1.25	0.162	0.078	0.212	1.25	0.650	0.009	1.431	1.25	0.243	0.056
Left CA3	4.237	1.25	0.51	0.150	0.347	1.25	0.561	0.014	0.986	1.25	0.331	0.039
Right CA3	2.680	1.25	0.115	0.100	0.011	1.25	0.916	0.000	1.376	1.25	0.252	0.054
Left subiculum	49.926	1.25	**0.001^∗∗∗^**	0.675	0.959	1.25	0.337	0.038	0.094	1.25	0.762	0.004
Right subiculum	12.976	1.25	**0.001^∗∗∗^**	0.351	2.025	1.25	0.168	0.079	0.001	1.25	0.976	0.000
Left CA4/DG	9.480	1.25	**0.005^∗^**	0.283	0.541	1.25	0.469	0.022	0.001	1.25	0.961	0.000
Right CA4/DG	0.002	1.25	0.963	0.000	0.595	1.25	0.448	0.024	2.32	1.25	0.141	0.088

*CA, cornu ammonis; DG, dentate gyrus; p ≤ 0.05 = statistical significance. Level of significance: 0.01 ≤ α < 0.05: ^∗^“significant”; 0.001 ≤ α < 0.01: ^∗∗^“high significant”; α < 0.001: ^∗∗∗^“highly significant”.*

### Postural Control

**FIGURE 5:**
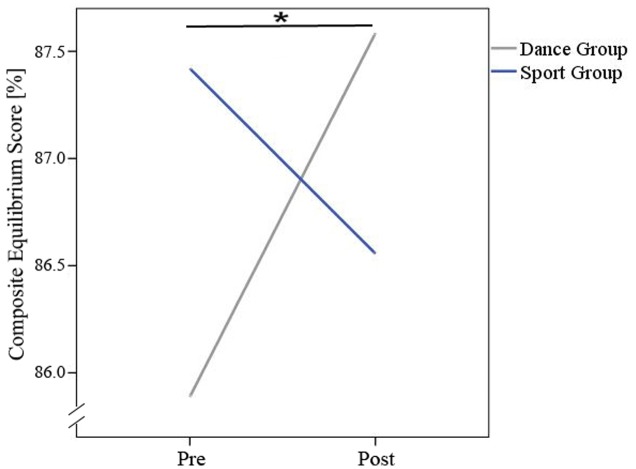
Interaction-effect (time × group) for composite equilibrium score. ^∗^Means significant interaction-effect.

Repeated measurement ANOVAs of balance data showed an interaction effect with group for the composite equilibrium score (see **Table 3** and **Figure 5**).

**Table 3:** Statistical values of repeated-measures ANOVAs for sensory organization of balance.

Sensory organization of balance [%]	Time				Group				Interaction (time × group)			
	*F*	df	*p*	η^2^	*F*	df	*p*	η^2^	*F*	df	*p*	η^2^
Somatosensory system	30.340	1,25	**0.001^∗∗∗^**	0.591	1.208	1,25	0.284	0.054	0.692	1,25	0.415	0.032
Visual system	6.094	1,25	0.022^∗^	0.225	0.363	1,25	0.553	0.017	0.296	1,25	0.592	0.014
Vestibular system	17.722	1,25	**0.001^∗∗∗^**	0.458	0.229	1,25	0.637	0.011	1.326	1,25	0.262	0.059
Composite equilibrium score	0.514	1,25	0.481	0.024	0.092	1,25	0.764	0.004	4.851	1,25	0.039^∗^	0.188

*Level of significance: 0.01 ≤ α < 0.05: ^∗^“significant”; 0.001 ≤ α < 0.01: ^∗∗^“high significant”; α < 0.001: ^∗∗∗^“highly significant”.*

There was a main effect of time regarding the somatosensory and vestibular contribution but no significant time × group interaction effects after 18 months of training (see **Table 3**). *Post hoc* tests revealed that the dancers improved in the use of all three sensory systems somatosensory system [*t*(13) = -2.902, *p* = 0.004], visual system [*t*(12) = -2.525, *p* = 0.027] vestibular system [*t*(12) = -3.271, *p* = 0.007] to maintain balance. Members of the sports group improved in the use of the somatosensory system [*t*(9) = -3.579, *p* = 0.006] and the vestibular system [*t*(9) = -3.881, *p* = 0.004] but not in the visual system. **Table 3** presents an overview of significant alterations related to employment of sensory information to maintain balance from baseline to post-intervention for both groups.

### Correlation Analysis

Correlation analysis between all hippocampal subfields and balance did not yield any significant results irrespective of whether the groups were analyzed separately or jointly.

## Discussion

Animal research has shown that combining aerobic training with sensory enrichment has a superior effect on inducing neuroplasticity in the HC compared to physical exercise or sensory stimulation alone (Kempermann et al., 2010). This sparked our idea to investigate the impact on neuroplasticity in elderly humans of a specially designed, sensorimotor and cognitive challenging dance program in comparison to a classical cardiovascular fitness program. In addition to our previous work (Müller et al., 2017), in the present study we ran a dedicated ROI analysis, which was focused on subfield volumes of the HC. The HC is of special interest as this brain structure is (a) especially affected by normal and pathological aging and (b) plays a key role in major cognitive processes, e.g., memory and learning and (c) is also involved in keeping one’s balance, a function which is crucial for well-being and quality of life.

We observed that both, dancing and fitness training led to increases in hippocampal subfield volumes. Although there was no significant group × time interaction in the ANOVA omnibus analysis, exploratory *post hoc* *t*-tests indicated that participants of the dance group showed volume increases in more subfields (four out of five, including the DG) of the left HC and that only dancing led to an increase in one subfield of the right HC, namely the subiculum. Regarding balance abilities dancing was superior to standard fitness as expressed by a larger increase in the composite score of our balance test and improved use of all three sensory systems. We, however, did not observe a correlation between changes in HC subfield volumes changes and those in balance; in other words whether the observed skill improvement can be attributed to the HC cannot be fully answered yet.

Regarding the HC volume increases observed in both groups, our results support the assumption that HC volume can be enhanced by physical fitness alone, as this was the overlapping feature of both trainings. Animal studies have shown that adult neurogenesis takes place mainly in the DG part of the HC (van Praag et al., 1999). Interestingly, only the dancers showed an increase in this brain region. Whether adult neurogenesis was indeed the basis of the here observed volume change, however, must remain an open question as there is no direct way in addressing this process in humans.

The dancers showed increases in some HC subfields where there was no change to be observed in the sports group. This indicates that apart from physical fitness, other factors inherent in dancing, contribute to HC volume changes, too. Animal research has suggested that sensory enrichment may be such a factor whereby physical fitness and enrichment have different effects on HC neurons: running in a wheel generates new neurons in the HC of mice but these only survive when sensory stimulation is also present (Kempermann et al., 2010). Again, with our own data we cannot differentiate between these different processes. We nevertheless can conclude that the additional challenges involved in our dance program, namely cognitive and sensorimotor stimulation, induced extra HC volume changes in addition to those attributable to physical fitness alone. It is noteworthy, that other studies in elderly humans, which did not boost physical fitness but which were sensorimotor demanding, such as learning to juggle (Boyke et al., 2008), have observed HC volume increases as well.

Only the dancers showed an increased balance composite score and they improved in all three involved sensory systems. This indicates that dancing drives all three senses and presumably also improves the integration of sensorimotor, visual and vestibular information. Balancing is an important everyday function, crucial for example for social mobility. Impaired balance often results in falls, which constitutes a major health risk factor with consequences both on morbidity (and even mortality) and health care costs (see also Dordevic et al., 2017). Although the ability to balance has been also linked to the HC and its connections, for example, to the vestibular system (Brandt et al., 2005), we did not observe a correlation between HC subfield volumes and improvements in balance. Given the small size of our sample, this needs to be interpreted with care but may suggest that other brain regions, probably those described in our earlier analysis (Müller et al., 2017) were involved in these improvements or that changes in the HC other than those expressed in measurable volumes, e.g., synaptic function, perfusion, etc. contributed to this effect.

There are other limitations in the present study which should not be left unmentioned. As already mentioned above (see Materials and Methods), we had to change the training intervention frequency from twice a week to once a week after 6 months of training. Hence, it must remain unclear whether more pronounced effects could have been observed if we had been able to stick to the initial training intensity. Next the ANOVAs failed to reach significant group interaction effects, only the exploratory *t*-tests became significant which may be a consequence of the large number of factors and levels in the ANOVAs on the one hand, and the small sample size on the other hand. A further limitation can be seen in the use of fully automated segmentation tools. Finally, the small sample size accompanied by a high drop-out rate as well as the highly selective inclusion, a missing inactive control group and exclusion criteria must be mentioned as they limit the generalizability of our results.

In sum, the present results indicate that both dance and fitness training can induce hippocampal plasticity in the elderly, but only dance training improved balance capabilities.

However, larger studies with more representative samples are required in the future. They should include additional analysis of mediating factors and they should try to find ways to optimally adjust the training protocol to an individual’s needs and preferences. Most of all, it needs to be investigated in longitudinal randomized clinical trials whether the proposed interventions indeed have the potential to reduce or postpone the risk of neurodegenerative diseases such as Alzheimer’s as suggested in large non-interventional studies (Verghese et al., 2003).

## Author Contributions

KR was responsible for the study organization and execution, as well as writing the text of the manuscript (Introduction, Discussion, and some parts of the Materials and Methods: Study Design and Subjects). PM contributes equally to this work. He has written some parts of the manuscript (Materials and Methods, Results, Discussion). NA assessed balance abilities and analyzed the data. He has written some parts of the Materials and Methods and Results (postural control). MS contributes to the Statistical Analysis and did some corrections of this manuscript. MD supported hippocampal subfield analysis and corrected this manuscript. JK contributes to the MRI measurements and for structural brain analysis. He corrected this manuscript. AH is the chief coordinator of this study and selected the motor skill tasks and organized the framework. NM is the second chief coordinator of this study and provided MRI measurements. He also worked on the Introduction and Discussion of this manuscript.

## Conflict of Interest Statement

The authors declare that the research was conducted in the absence of any commercial or financial relationships that could be construed as a potential conflict of interest. The reviewer MT declared a shared affiliation, though no other collaboration, with several of the authors KR, NA, MD, JK and AH to the handling editor, who ensured that the process met the standards of a fair and objective review.
